# Association of serum uric acid level with mortality and morbidity of patients with acute ST-elevation myocardial infarction

**DOI:** 10.15171/jcvtr.2016.11

**Published:** 2016-06-28

**Authors:** Reza Hajizadeh, Samad Ghaffari, Rezvanieh Salehi, Sarvin Mazani, Sharmin Aghavali

**Affiliations:** ^1^Cardiovascular Research Center, Tabriz University of Medical Sciences, Tabriz, Iran; ^2^Faculty of Medicine, Tabriz University of Medical Sciences, Tabriz, Iran

**Keywords:** Acute Myocardial Infarction, Serum Uric Acid, Left Ventricular Systolic Dysfunction, Cardiac Troponin I

## Abstract

***Introduction:*** Investigating the clinical impact of serum uric acid (UA) and its lowering agents on the complications and mortality of acute ST-elevation myocardial infarction (STEMI) can open a new era in STEMI treatment. The aim of this study was to evaluate the effect of on admission serum UA level on the mortality and morbidity of patients admitted with STEMI.

***Methods:*** A number of 608 patients with STEMI were enrolled in this study from December 21, 2012 until February 19, 2014. Patients were followed for 20 months. Male to female ratio was 2.53, and the mean age of patients was 62.6±13.4. The relationship between the level of UA and patients’ mortality and morbidity, left ventricular ejection fraction (LVEF), atrial and ventricular arrhythmia was analyzed.

***Results:*** Patients with high serum UA level had higher Killip class after STEMI (*P*=0.001). Mean LVEF was measured to be 39.5±9.6 in normal UA group and 34.6±11.6 in high UA group (*P*=0.001). In comparison with normal UA group, high UA group had significantly higher cTnI (2.68±0.09 vs 4.09±0.42, respectively, *P*=0.001), increased blood pressure (*P*=0.009), and higher atrial fibrillation (AF) occurrence (*P*=0.03), but no association was seen between ventricular tachycardia and serum UA level. Short term and midterm mortality were not different in two groups (*P*=0.44 and 0.31, respectively).

***Conclusion:*** In the current study, high serum UA level in patients with acute myocardial infarction (MI) was not associated with higher in-hospital or midterm mortality, but it was associated with lower LVEF, higher Killip class, elevated cTnI, creatinine, triglyceride, and higher AF.

## Introduction

Ischemic heart disease (IHD) with 7.4 million deaths and hypertension with 1.1 million deaths per year have been a major cause of mortality during the past decade.^1^ Recent investigations have shown a high incidence of all cardiovascular diseases especially MI in Iran.^2,3^ Better understanding of the pathophysiology of disease and prognostic biomarkers can result in better treatment of patients.

Intra-coronary thrombosis formation can cause coronary artery blood flow occlusion, which results in MI. Irreversible myocardial damage and cell death would occur if occlusion of coronary artery lasts more than 20 minutes.^4^

Adenosine produced by cardiac myocytes causes the vasodilatation of arteries. The level of adenosine is increased under some conditions such as hypoxia and tissue ischemia.^5^ Endothelium degrades adenosine to uric acid (UA) rapidly; so due to increasing the concentration of UA and decreasing the intracellular PH, rapid UA efflux to the vascular lumen occurs.^6^ hence, UA level may be an indicator of severity of ischemia and tissue hypoxia and high serum UA has been stipulated as a risk factor for coronary artery disease (CAD) and somehow as prognostic factor for mortality and morbidity in patients with CAD. Chen et al showed association between serum UA and adverse events in patients with STEMI.^7^ Kroll et al showed that in patients with higher on admission serum UA, short term and long term mortality was increased.^8^ In other study done in Iran higher serum UA had association with in-hospital and short term mortality.^9^ By developing new treatments especially increasing trend to invasive therapy in high risk patients for example those who develop heart failure after ST-elevation myocardial infarction (STEMI) and continuous effort to decrease STEMI mortality, it is interesting to investigate whether prognostic factors such as serum UA continue to have their prognostic value or not.

In this study, we investigated on admission serum UA levels as a potential predictor of in-hospital and 20 months follow up period mortality and morbidity in patients with MI.

## Patients and Methods

### Study design and setting

The aim of this cross-sectional study was to evaluate the effect of on admission serum UA on the short term and mid-term mortality and morbidity of patients admitted with STEMI.

We studied 608 patients admitted from December 21, 2012 till February 19, 2014 at Shahid Madani hospital, Tabriz, Iran. Patients were divided into two groups according to their serum UA level.

### Inclusion and exclusion criteria

Patients with STEMI were enrolled in this study. Acute MI was defined as presence of ST-segment elevation consistent with the MI at least 2 mm in adjacent chest leads and/or ST-segment elevation at least 1 mm in 2 or more standard leads or new left bundle branch block, concordant with positive cardiac necrosis markers.^10^

Hypertension was interpreted as blood pressure ≥140/90 or taking anti-hypertensive medications.^11^ Having fasting blood glucose level higher than 126 mg/dL or having a random blood glucose test level of 200 mg/dL or higher as well as those taking medications for hyperglycemia were defined as diabetes.^12^ Hyperlipidemia was defined as cholesterol level ≥200 mg/dl and triglyceride ≥250 mg/dl.^13^

Patients with liver disease, progressive kidney disorders (creatinine >1.8), gout, alcoholism or taking antihyperuricemic drugs were excluded. Patients with previous history of diuretic and losartan use, also patients with previous history of MI, were excluded.

### Study protocol

Blood samples were taken as soon as possible after admission. Laboratory data was gathered and UA level measurement was performed according to the standard procedures of our laboratory at Madani heart hospital, Tabriz, Iran. High UA level was defined as more than 8 mg/dL in men and more than 7.5 mg/dL in women.^14^ Transthoracic echocardiography was used to evaluate LVEF.

### Statistical analyses

SPSS version 17.0 was used for statistical analysis. Continuous data were expressed as mean ± SD/SE. The *t* test was used to compare continuous variables and Chi-square test was used to compare qualitative variables. UA and lipid profiles relationships were calculated with Pearson correlation coefficients. *P* value <0.05 was considered significant.

With a sample size of 608 patients and based on previous studies^7^ the statistical power of this study calculated to be 90%.

## Results

**Figure 1:**
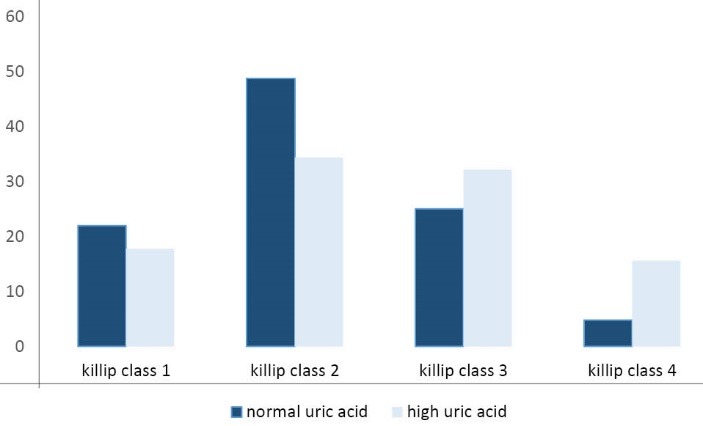
Killip class of patients according to their serum UA level.

In this cross-sectional study, we enrolled 608 patients with definite diagnosis of STEMI. Male to female ratio was 2.53, the mean age of patients was 62.6 ± 13.4, and patients were divided into two groups according to their serum UA level. Table 1 shows the demographic, medical, and drug history of patients according to their serum UA level. Patients with high UA had higher Killip class after STEMI (*P *= 0.001). Figure 1 shows the Killip class differences in two groups. Regarding the STEMI risk factors, smoking was more common in normal UA group (*P *= 0.02), but Increased blood pressure was seen more frequently in high UA group (*P *= 0.009), and diabetes mellitus and cholesterol level were not different in two groups (*P *= 0.57 and 0.81, respectively).

**Table 1:** Demographic, medical, and drug history of patients according to their serum UA level

	**Total** **608 (100%)**	**Low UA group** **518 (85.2%)**	**High UA group90 (14.8%)**	***P*** ** value**
Age	62.6±13.4	61.8±13.4	67.5±12.4	0.001
Gender, male	436 (71.7%)	378 (72.9%)	58 (64.4%)	0.097
Familial history of CAD	85 (13.9%)	78 (15.0%)	7 (7.7%)	0.066
Antihypertensive drugs	267 (43.9%)	216 (41.7%)	51 (56.6%)	0.008
Antidiabetic drugs	117 (19.2%)	96 (18.5%)	21 (23.3%)	0.286
Opiate	57 (9.3%)	51 (9.8%)	6 (6.6%)	0.340
Stroke	15 (2.4%)	10 (1.9%)	5 (5.5%)	0.056
Renal failure	30 (4.9%)	17 (3.2%)	13 (14.4%)	0.001

In comparison with normal UA group, high UA group had significantly higher cTnI (2.68 ± 0.09 vs 4.09 ± 0.42, respectively with a range of 0-14, median=2, and *P *= 0.001) and higher creatinine level (*P *= 0.001). Table 2 shows the paraclinical values of patients according to their serum UA concentrations. Mean LVEF was measured 39.5 ± 9.6 in normal and 34.6 ± 11.6 in high UA group (*P *= 0.001).

**Table 2:** Comparison of paraclinical values of patients according to their serum UA level

	**Total 608 (100%)**	**Low UA group** **518 (85.2%)**	**High UA group** **90 (14.8%)**	***P*** ** value**
Cholestrol >200 mg/dL	212 (34.9%)	184 (35.5%)	30 (33.3%)	0.811
Cholesterol value*	237.3±30.1	238.1±29.7	232.5±32.3	
TG >250	53 (8.7%)	39 (7.5%)	14 (15.5%)	0.023
TG value*	381.4±121.6	366.2±113.0	423.6±138.7	
CPK**	693.0±57.6 (median:259)	625.2±41.1 (median: 256)	1104.0±319.9 (median: 341)	0.001
CPK-MB**	92.3±6.8 (median:52.5)	90.0±7.5 (median: 52)	102.3±16.4 (median:55.5)	0.001

Abbreviations: TG, triglycerides; CPK, creatine phosphokinase. *Mean ± standard deviation, ** mean ± standard error.

We found that serum UA level was associated with higher AF occurrence (*P *= 0.03), but no association was observed between the serum UA level and ventricular tachycardia. Short term and midterm mortality (average follow up was 20 months) were not different between two groups (*P *= 0.44 and 0.31, respectively). Table 3 shows the complications and management of patients during the hospital course.

**Table 3:** Complications and management of patients during the hospital course

	**Total** **608 (100%)**	** Low UA group** **518 (85.2%)**	**High UA group** **90 (14.8%)**	***P*** ** value**
Thrombolytic therapy	215 (35.3%)	187 (36.1%)	28 (31.1%)	0.361
Primary PCI	453 (74.5%)	402 (77.6%)	51 (56.6%)	0.001
VT/VF	29 ‏)4.7%)	24 (4.6%)	5 (5.5%)	0.602
In-hospital death	38 (6.2%)	34 (6.5%)	4 (4.4%)	0.443

Abbreviations: PCI, percutaneous coronary intervention; VT, ventricular tachycardia; VF, ventricular fibrillation; UA, uric acid.

## Discussion

Some studies have shown that in patients with MI, high serum UA level can increase the mortality rate.^7,15,16^ In the current study, we could not find this association and according to patients’ serum UA level, in-hospital mortality and midterm mortality were not different between two groups. Kojima et al divided patients according to their serum UA level into four groups. Patients whose serum UA level was more than 399 µmol/L had 3.7 times higher total mortality rate than those with a serum UA level below 274 µmol/L. In this study, mean age of patients (67 years old) was higher than that of our study (62 years old).^15^ In addition, previous studies revealed that high serum UA level in patients with MI accompanied poor clinical prognosis such as heart failure, low LVEF, and high LV end diastolic diameter (LVEDD).^15,17^

In epidemiologic studies, high UA level has been established as a risk factor for the cardiovascular disease.^18,19^ In this study, we showed the association between high UA level and serum Cr and triglyceride levels. Tuomilehto et al and Nagahama et al reported similar results.^20,21^ Nagahama et al showed positive correlation between increased serum UA level and obesity, increased blood pressure and dyslipidemia.

Also Chen et al showed a significant association between UA level and dyslipidemia.^7^ In this study, no correlation was found between high serum UA level and hypercholesterolemia.

We found that higher UA level had association with higher AF occurrence. Similarly Huang et al and Tamariz et al reported higher UA level in patients with AF rhythm.^22,23^ Huang et al showed that in patients with AF who had not obstructive CAD, coronary blood flow had independent association with serum UA level.

Pozzoli et al showed that the onset of AF in patients with heart failure and sinus rhythm was associated with significant worsening in patients’ NYHA functional class.^24^ Maisel et al showed that incidence of AF was increased significantly in patients with more severe heart failure.^25^ However, we did not evaluate the frequency of arrhythmias using Holter monitoring and our arrhythmia evaluation was focused on sustaining the symptomatic arrhythmias.

In this study a significant correlation was found among high serum UA level, low LVEF, and higher Killip class, and Patients with high serum UA level were older, and had higher creatinine. These factors may be responsible for lower percutaneous coronary intervention (PCI) therapy and subsequently higher Killip class in these patients. There are other hypotheses about how high UA level is associated with low LVEF. One of these mechanisms focuses on the impact of UA on the efficacy of thrombolysis. Purine catabolism produces UA by the act of xanthine oxidase enzyme. UA is released in hypoxic conditions.^26^ In patients who develop heart failure after MI, tissue hypo perfusion and hypoxia lead to the activation of xanthine oxidase and other oxidative products.^27,28^ Viña et al reported that the xanthine oxidase is responsible for free radical production. They showed that tissue damage can be caused by the xanthine oxidase activity during heavy exercise. In their study allopurinol could reduce the oxidation of glutathione.^29^ Moreover, free radicals might be responsible for the inefficacy of re-perfusion therapy. Serum UA level can predict coronary blood flow in patients with acute STEMI treated with primary PCI.^30^ Okada et al reported that IL-1 induced free radicals stimulate PAI-1 in rats. In addition, they suggested that inhibition of fibrinolysis may be responsible for the serious cardiac dysfunction after the ischemia.^31^ However, the exact effect of fibrinolysis on the free radicals production is not clearly understood.

Kaya et al showed that elevated serum UA level on admission was an independent factor of short term and long term adverse events in patients with STEMI who were treated with primary percutaneous angiography.^32^

Evaluating the efficacy of some medications to reduce oxidative stress (e.g., xanthine oxidase inhibitors) after thrombolytic therapy may be helpful and we need large prospective studies to evaluate the effect of serum UA lowering agents in reducing adverse events in patients who develop acute STEMI.

These findings suggest that, serum UA level can be used as a prognostic factor in MI patients.

## Limitations

This study had some limitations. We did not have 24-hour electrocardiogram (ECG) monitoring and our data only consisted of sustained AF and VT. Because of small number of patients who developed arrhythmia, more investigations with larger sample size can help in better evaluation of association of AF and VT and serum UA level. Finally because IHD is a chronic disease, long term follow-up is needed for better evaluation of long term effects of UA on the cardiovascular system.

## Conclusion

In the current study, high serum UA level in patients with acute MI was not associated with higher in-hospital or midterm mortality rate. However, high serum UA level was associated with lower LVEF, higher Killip class and AF, elevated cTnI, creatinine, and triglyceride level.

## Acknowledgments

We would like to thank all the patients, nurses, and doctors of Shahid Madani heart center.

## Ethical Approval

This study approved by the local ethical committee.

## Competing of interests

None to be declared.
